# Parasites and Wildlife

**DOI:** 10.3390/ani13040628

**Published:** 2023-02-10

**Authors:** Rafael Calero-Bernal, Ignacio García-Bocanegra

**Affiliations:** 1 Salud Veterinaria y Zoonosis (SALUVET), Department of Animal Health, Faculty of Veterinary, Complutense University of Madrid, 28040 Madrid, Spain; 2 Grupo de Investigación en Sanidad Animal y Zoonosis (GISAZ), Departamento de Sanidad Animal, UIC Zoonosis y Enfermedades Emergentes ENZOEM, Universidad de Córdoba, 14014 Córdoba, Spain

Macro and micro-parasites are integrated into ecosystems worldwide and are considered important elements of biodiversity. Close relationships between parasites and vertebrate hosts cause evolutionary changes in each, which are sometimes driven by the heterogeneity of elements composing the ecosystems. Knowledge of parasites affecting human, domestic, and wild animal species requires, therefore, a deep understanding of the complex network created by the interactions between these pathogens, host species, and ecosystems. Nowadays, researchers face the challenge of studying the unpredictable change of these interactions as consequences of natural and anthropic disequilibrium.

In a changing world, most transboundary diseases have been propagated from the wilderness to anthropized areas. During the last decades, societies are increasingly concerned about the relevance of wildlife as a fundamental part of Global Health. In this respect, research on parasites that may affect the biology and population equilibrium of wildlife is of major interest, especially when a One Health perspective is considered.

The present Special Issue “Parasites and Wildlife” was aimed at providing a collection of comprehensive investigations falling within a number of areas of interest such as epidemiology, diagnosis, emerging zoonoses, food safety, conservation issues, parasite–host interactions, and pathology in infections caused by parasites in wild host species.

In sum, eight articles, two communications, and two brief reports from over 103 authors based in field areas of nine countries have been compiled. The pretended international dimension of the proposal was achieved given the fact that 44 institutions from 18 countries were involved (Figure 1a). Investigations were focused on 51 wild, including avian and mammal, host species and were focused on several topics represented by the keywords summarized in Figure 1b.

An overview of the research compiled according to the taxonomic organization and complexity of the target organisms (fungi, protozoa, helminths, and arthropods) is presented below.

In recent years, investigations on protozoan parasites have significantly increased and this is reflected in the present collection. Köster et al. [1] investigated, by means of molecular methods, the occurrence and genetic diversity of intestinal and blood protozoa as well as filariae in faecal samples from wild chimpanzees (*Pan troglodytes verus*) in the Dindefelo Community Nature Reserve, Senegal. Findings suggested that chimpanzees might play a more complex role in the epidemiology of pathogenic and commensal fungal, protozoan, and nematode species than initially anticipated, and two important facts contributed to the interest of such research: (i) wild chimpanzee populations in West Africa have dramatically decreased as a direct consequence of anthropogenic activities and infectious diseases, and (ii) findings suggested potential cross-species transmission between wild chimpanzees and humans in areas where both species overlap. Indeed, the presence of zoonotic Microsporidia was investigated by Martínez-Padilla et al. [2] in kidney samples of European wild rabbit (*Oryctolagus cuniculus*) and Iberian hare (*Lepus granatensis*) consumed by humans in Spain. Molecular methods allowed the detection of *Enterocytozoon bieneusi* and *Encephalitozoon intestinalis* but not *E. hellem* nor *E. cuniculi*. Despite the fact that the presence of the above agents may pose a public health concern, additional studies are required to define the frequency and characterize the real potential associated with zoonotic risk.

The group of Apicomplexan parasites have been extensively addressed in this Special Issue. Prakas et al. [3] searched for the role of mustelids as definitive hosts in the transmission of various *Sarcocystis* spp. in Lithuania. Intestinal scraping of five species of mustelids was examined by molecular methods and four species of *Sarcocystis* including the zoonotic *S. hominis* were detected. The study provided strong evidence that members of the Mustelidae family serve as definitive hosts for *Sarcocystis* spp. using cattle as intermediate hosts and raising important connections between carnivore wildlife and livestock. In a similar line, Giorda et al. [4] investigated the presence of tissue cysts of an acknowledged *Sarcocystis*-like organism in the brain and muscle of two stranded striped dolphins (*Stenella coeruleoalba*) in the Ligurian coast of Italy. Non-suppurative meningoencephalitis was associated with the presence of genetic variants that showed the highest homology to *Sarcocystis* spp. infecting the Bovidae family. The present study added valuable information on the complex epidemiology of the Sarcocystis genus. Evidence of sarcocystid infection was also investigated by Acosta et al. [5] in three species of seabirds from Magdalena Island, Chile. Findings of ITS1, 18S, and cox1 nucleotide sequences from muscle tissues of two skuas, revealed closely related homologous sequences of *Sarcocystis halieti*, that is a species found in seabirds of the northern hemisphere. Further studies are needed to understand the epidemiology of the infection and its impact on the health of marine fauna.

Barroso et al. [6] carried out a serosurvey to investigate the potential environmental and host-dependent variables that may affect the prevalence of antibodies of the zoonotic protozoan *Toxoplasma gondii* in wild boar (*Sus scrofa*), red deer (*Cervus elaphus*), and fallow deer (*Dama dama*) present in the Doñana National Park, Spain. The high seroprevalence values detected in the three wild ungulate species analyzed suggested that the complex interplay of hosts and eco-epidemiological niches, along with the optimal climatic conditions for the oocysts’ survival, may favor the spread of the parasite in its host community in the study area. The concomitance of effects among the species indicated that relevant drivers of risk operated at the community level.

The zoonotic character and the complex epidemiology of some helminthic genuses motivates continuous research. In this context, Skrzypek et al. [7] investigated, by means of molecular methods, the presence of the important zoonotic *Echinococcus multilocularis* cestode in fecal samples of red foxes (*Vulpes vulpes*) from Poland. Two DNA extraction methods were compared aiming at improving the sensitivity of the diagnostic assay. High prevalence figures detected indicated the importance of the host in the life cycle of the parasite. In addition, these figures implied that both extraction methods showed similar efficiency in DNA isolation and dealing with inhibitors. The number of worms detected in the intestines had no influence on the PCR results. Additional aspects related to the degradation of genetic material potentially harming the reliability of the diagnostic methods used on field-collected samples were highlighted.

Land-use changes are one of the most important drivers of zoonotic disease risk in humans, including parasites of wildlife origin. The effect of this anthropogenic land-use change on the parasitism (presence and prevalence) of intestinal helminths in wild rodents was investigated by Riquelme et al. [8] in central Chile. Despite the fact that the overall helminth prevalence was 16.95%, and some zoonotic species (*Hymenolepis* spp.) were present, the effect of habitat type, native forests, and adult and young pine plantations, the prevalence was not observed, while other factors, such as rodent species and season of the year, were relevant to explain changes in helminth prevalence. Therefore, additional investigations focused on habitat alterations and potential changes in parasitofauna are encouraged. Kärssin et al. [9] carried out a survey on the presence of *Trichinella* spp. in four wild free-ranging host species, including wild boars, brown bears, Eurasian lynxes, and badgers, in Estonia. All four European *Trichinella* species, including non-encapsulated *T. pseudospiralis*, were detected, and results indicated high infection pressure in the sylvatic cycles across the years—illustrating the continuous risk of spillover to domestic cycles and of transmission to humans.

Currently, the evolving arthropod populations and their impact as disease vectors are undeniable. Yin et al. [10] investigated the potential host selection of the tropical rat gamasid mite (*Ornithonyssus bacoti*) on different animal hosts and the distribution in different environmental gradients in Yunnan Province of Southwest China. Different parameters including mite abundance were evaluated in 15 host species including rodents and other small mammals. In sum, the main reservoirs of *O. bacoti* were the synanthropic rat species, *Rattus tanezumi*, and *R. norvegicus*, and along with the observed major distribution in the flatland landscape and indoor habitats raises important questions on the impact on the public and on the health of animals, as well as the interconnections of the sylvatic and domestic life cycles of the mite. Two studies approached the occurrence of vector-borne pathogens in mammal wild hosts; Hornok et al. [11] molecularly screened for vector-borne protozoan parasites and bacteria potentially present in liver tissues of the only native terrestrial mammal in Iceland, the arctic fox (*Vulpes lagopus*). In this pioneering study in Europe, and considering the importance of the vector tick species *Ixodes ricinus*, only DNA of *Anaplasma phagocytophilum* was detected. Results provide valuable information as baseline data for comparison in the tfuture monitoring of the emergence of ticks and tick-borne diseases, especially when considering the incoming warming climate and the predictable increase of the presence of *I. ricinus* in the area. In addition, Mardosaitė-Busaitienė et al. [12] investigated the role of the reservoir host of eight species of rodents for emerging *Babesia microti* in Lithuania. The remarkable prevalence of *B. microti* was detected in the species investigated and parasites identified presented molecular homology with zoonotic strains; in addition, a comprehensive ecological study allowed the identification of a gradient of prevalences in different habitats compatible with the sustainability of the life cycle.

Overall, the papers in this Special Issue reveal different perspectives of current research on parasitic diseases and their relationship with the wildlife compartment; it is clear that it is necessary to continue these field-based studies to unravel the intricate interactions between pathogens and wildlife, livestock, and humans, confirming the three compartments of the One Health approach.

## Figures and Tables

**Figure 1:**
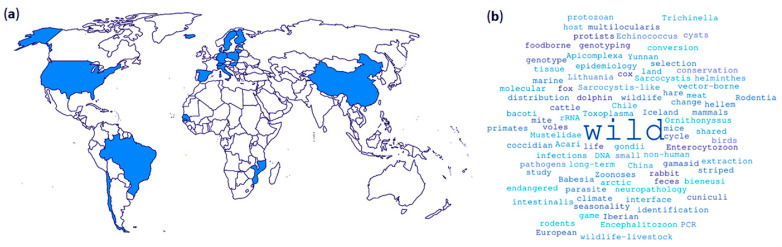
(**a**) Worldwide research institutions (*n* = 44) from 18 countries participating in the interdisciplinary Special Issue on Parasites and Wildlife. (**b**) Word cloud with the keywords utilized by each research paper. Brazil (*n* = 2): University of São Paulo, University Santo Amaro; Chile (*n* = 2): Universidad de Chile, Universidad de Concepción; China (*n* = 3): Guizhou University, Provincial Key Laboratory for Agricultural Pest Management in Mountainous Region, Dali University; Denmark (*n* = 3): University of Copenhagen, Technical University of Denmark, Statens Serum Institut; Estonia (*n* = 2): Estonian Veterinary and Food Laboratory, Estonian University of Life Sciences; Finland (*n* = 1): University of Helsinki; Germany (*n* = 2): Leibniz Institute for Zoo and Wildlife Research, Freie Universität Berlin; Hungary (*n* = 2): University of Veterinary Medicine, Centre for Agricultural Research; Iceland (*n* = 1): Icelandic Institute of Natural History; Italy (*n* = 5): Istituto Zooprofilattico Sperimentale del Piemonte, Liguria e Valle d’Aosta, Local Veterinary Services in Imperia, University of Siena, University of Teramo, Local Veterinary Services in Asti; Lithuania (*n* = 2): Vytautas Magnus University, Nature Research Centre; Mozambique (*n* = 1): Universidade Licungo; Poland (*n* = 1): National Veterinary Research Institute; Senegal (*n* = 1): Jane Goodall Institute Senegal; Spain (*n* = 11): Institutes of Health Carlos III, Jane Goodall Institute Spain, Complutense University of Madrid, University of Las Palmas de Gran Canaria, Mundomar, Universidad de Córdoba, Universidad de Castilla-La Mancha, Consejo Superior de Investigaciones Científicas, Universidad de Málaga, Universidad San Pablo-CEU, Junta de Andalucía; Sweden (*n* = 1): Lund University; Switzerland (*n* = 1): University of Zurich; USA (*n* = 3): Ohio State University, Department of Health and Human Services, Food and Drug Administration, United States Department of Agriculture.
